# Molecularly Imprinted Polymers for the Selective Extraction of Bisphenol A and Progesterone from Aqueous Media

**DOI:** 10.3390/polym10060679

**Published:** 2018-06-19

**Authors:** César Cáceres, Catalina Bravo, Bernabé Rivas, Ewa Moczko, Pedro Sáez, Yadiris García, Eduardo Pereira

**Affiliations:** 1 Departamento de Polímeros, Facultad de Ciencias Químicas, Universidad de Concepción, Edmundo Larenas #129, Concepción 4070371, Chile; cecaceres@udec.cl (C.C.); brivas@udec.cl (B.R.); 2 Departamento de Química Analítica e Inorgánica, Facultad de Ciencias Químicas, Universidad de Concepción, Edmundo Larenas #129, Concepción 4070371, Chile; labravo@udec.cl (C.B.); ygarcia@udec.cl (Y.G.); 3 Departamento de Química Ambiental, Facultad de Ciencias, Universidad Católica de la Santísima Concepción, Alonso de Rivera #2850, Concepción 4090541, Chile; ewa@ucsc.cl; 4 Sección Microanálisis, Laboratorio de Criminalística Central, Policía de Investigaciones de Chile PDI, Carlos Silva Vidósola #9783, La Reina, Santiago 7860379, Chile; psaezm@investigaciones.cl

**Keywords:** molecularly imprinted polymers, endocrine disruptors, selective extraction

## Abstract

This paper describes the development of a novel sorbent for selective extraction of endocrine disruptors (EDs) from aqueous media. The main goal was to obtain sufficient molecularly imprinted polymers (MIPs) for selective detection, preconcentration, and extraction of EDs such as bisphenol A (BPA) and progesterone (PG). Series of MIPs and their analogues, non-molecularly imprinted polymers (NIPs), were synthesised following a non-covalent imprinting strategy based on radical polymerisation. Sets of synthesis were performed in order to optimise variables of the polymerisation including solvent, cross-linker, and template ratio. The retention capacity of MIPs was determined using HPLC in the range of 33.3% to 96.6% and 32.5% to 96% for BPA and PG, respectively. The adsorption mechanism was studied by isothermal and kinetic assays. The kinetic analysis showed a high retention capacity within 15 min of contact. The polymer yield was obtained in the range of 30% to 100%. Additionally, there was no significant cross-reactivity observed upon testing MIPs with structural analogues and other endocrine disruptors instead of target molecules. The results also revealed the high importance of different concentrations of cross-linker and solvent during the polymerisation. Firstly, the pre-organisation of complementary functional groups, which were present in the polymerisation mixture, and secondly, selective cavity formation for target molecules.

## 1. Introduction

The majority of vital functions of the human body can be altered by endocrine disruptors (EDs), which interfere with the natural production, release, or elimination of hormones. Therefore, they disturb the performance of the endocrine system and can cause adverse health effects in humans and other mammals [1,2,3,4]. It has been observed that particularly during fetal development and early childhood, exposure to low doses of EDs (ppb) can have serious effects on human life and reproduction, causing decreased mental capacity, genital abnormalities, and cancers [5,6]. Many EDs such as phenolic endocrine disruptors can often be found in rivers and ground waters as they are widely used in the plastic industry and regularly released to the environment. Further, they can migrate closer, contaminating food and drinking water [4,7,8]. Therefore, it is extremely important to develop a suitable technique that enables the fast and easy detection of low concentrations of EDs, particularly in aqueous media.

This article provides preliminary studies on the extraction and preconcentration of two important EDs, bisphenol A (BPA) and progesterone (PG). They have been identified as some of the most common hazardous substances in the human environment, often released from various plastics (e.g., baby or beverage bottles). Much evidence has shown that BPA has toxic properties, inducing estrogenic endocrine disruption and the acceleration of tumorigenic progression [9]. In 2018, the European Commission issued a new regulation on the use of BPA in packaging. The Specific Migration Limit (SML), which is the maximum amount of the chemical allowed to migrate from packaging into food, was lowered from 0.6 mg to 0.05 mg of BPA per kg of food. The new Tolerable Daily Intake (t-TDI) was set at 4 µg/kg body weight per day [10]. Additionally, since 2011, BPA has been forbidden in the manufacture of baby bottles and any food contact material intended for children up to 3 years of age. To protect aquatic life and mammalian consumers, the concentration of BPA in water has been set at 1.4 µg/L and in sediment at 9.9 µg/kg dry weight [11]. This has raised a great concern about the utilisation of BPA and its potential harmful effects on human health. Therefore, it is very important to monitor even trace amounts of BPA [12]. The major part of our work focused on the development of a new, sufficient method for the detection of BPA, which can further be applied in the determination of other highly dangerous EDs, e.g., progesterone (PG). PG is a hormone which stimulates and regulates the activity of cells and organs. Its secretion is controlled by the adrenal glands [13]. In the last 20–30 years, the knowledge about progesterone has significantly increased, leading to great progress in progestational therapy [14]. It has been reported that PG has revealed biological activity already at 123 ng/L, affecting human health, causing cancer, and disrupting natural hormonal activity. Currently, there are no laws that regulate the level of PG in the environment [15].

Over the years, scientists have been trying to develop new methods to detect EDs in environmental samples. Generally, the process involves lengthy pretreatment steps of the samples to reduce matrix interferences and to enhance the detection of the target hazardous compounds, particularly their trace amounts. Only after those steps it is possible to start proper extraction and analysis. Recently, the popular leading technique for the extraction of pollutants from environmental liquid samples has been Solid Phase Extraction (SPE), which allows relatively fast and easy measurements [16]. In the last decade, molecularly imprinted polymers (MIPs) combined with SPE have attracted much attention, especially in the extraction of trace amounts of analytes from large sample volumes and complex matrices. Moreover, MIPs are low-cost and very stable [17,18]. The principle of molecular imprinting is to create recognition sites for a target analyte in the polymeric matrix. During this process, functional and cross-linking monomers are polymerised or co-polymerised in the presence of an imprint molecule, called a molecular template [19]. During the synthesis, functional monomers form a complex with template molecule while the highly cross-linked polymeric backbone helps to keep functional groups in specific positions. After polymerisation, imprinted molecules are subsequently removed from the matrix, leaving cavities with an affinity to a chosen template. In that way, the polymer matrix keeps the molecular memory of the analytes and it can be used in their molecular recognition [20,21,22,23,24,25,26,27,28,29]. As a reference, non-molecularly imprinted polymers (NIPs) without the template are used, following the same synthesis procedure as for MIPs, except in the absence of a template. Therefore, NIPs have the same composition as MIPs but without the presence of any specific cavity. Therefore, the nature of the interactions developed between MIPs and the template is the same as those developed between NIPs and the template, while the difference between these two types of polymers is the strength of these interactions. If the cavities created during the polymerisation are well defined, the strength of the interactions in MIPs should be significantly better than those in NIPs. The retention properties of the synthesised polymers can be established based on the adsorption capacity and the mechanism of the adsorption of target analytes onto the surface of the polymeric material. One of the methods widely used to define these binding properties is the determination of adsorption isotherms. The Langmuir-Freundlich isotherms (LF) can model the adsorption of an adsorbate in MIPs and NIPs along with homogeneous and heterogeneous distributions in high and low concentrations of analytes [30,31].

MIPs have already proved their efficiency in selective preconcentration and measurements of endocrine disruptors in complex samples, such as ground water, milk, and serum [32,33,34,35]. In the detection of BPA, MIPs were used in several different formats, e.g., as a stationary phase in the capillary electrophoresis [31], superparamagnetic surface modified nanoparticles [36], or bulk materials combined with liquid chromatography-mass detection [37]. Despite many attempts of the synthesis and application of MIPs, they always suffered from low performance, complicated data analysis, and high batch variability. Similar problems were related to the separation and quantification of PG, e.g., using MIPs combined with SPE and HPLC [33], as hydrogels [38], or in the application of MIPs in electrochemical sensors, hydrogels [39,40], and optical biosensors [41]. Therefore, in our work we tried to enhance the synthesis and the performance of MIPs by the optimisation of different parameters during polymerisation, such as the concentration of the cross-linker, solvent, and template ratio. The preliminary work was performed in order to detect and quantify BPA and PG in water samples. Additionally, our objective was to contribute to the growing area of research involving imprinting polymers. Therefore, the purpose of the work was to use MIPs as a new highly efficient sorbent which could be applied in solid phase extraction. First part of the work involved testing a variety of functional monomers to select that most suitable for the polymerisation of the MIPs for BPA and PG (see Supplementary Materials). After that, it was necessary to perform a brief experimental design in order to optimise all variables that affect the process of the retention of target molecules, based on the maximum adsorption capacity using adsorption isotherms and kinetic studies.

The optimal designs were generated in MODDE software, which enabled us to screen and optimise experimental variables due to the integrated cross-validated model fitting, model fit visualisations, and predictive capabilities [42,43,44].

## 2. Materials and Methods

### 2.1. Materials

Reagents used in the experiments are listed as follows. Acrylic acid (AA), 1,4-divinylbenzene (DVB), 2,2′-azobisisobutyronitrile (AIBN), diosgenin (93%), and diphenylamine (99%) were purchased from SIGMA ALDRICH (St. Louis, MO, USA). Progesterone (PG 99%), bisphenol A (BPA, 98%), absolute ethanol (ETOH) grade P.A., absolute ethanol grade HPLC, acetic acid, acetonitrile P.A., and methanol HPLC were purchased from MERCK (Kenilworth, NJ, USA). Reagents and solvents were of analytical or higher grade and were used without further purification.

### 2.2. Methods

#### 2.2.1. Synthesis of Molecularly Imprinted Polymers (MIPs) and Non-Imprinted Polymers (NIPs)

The synthesis of MIPs was carried out after the preliminary experimental design was performed using MODDE 7.0 software (Malmö, Sweden). As result of computational analysis, five syntheses with different quantities of cross-linker, template, and solvent were generated, followed by two replicates of the last synthesis (see Table 1). The amounts of functional monomer (2.0 g) and initiator (2 mol % of the monomer) were kept constant through all experiments.

The appropriate amount of functional monomer (2.0 g AA) was weighed and placed in 50-mL Schlenk tubes, followed by the addition of a solvent (acetonitrile) and the template molecules (BPA or PG). The mixture was stirred for 5 min to obtain a homogeneous solution and left for 30 min without agitation. After that, the appropriate volume of cross-linker 1,4-divinylbenzene (DVB) was added into each tube and stirred for 5 min. The initiator 2,2′-azobisisobutyronitrile (AIBN) was dissolved in the trace amount of ethanol and added to the polymerisation mixtures. To displace all of the dissolved oxygen, the solutions were bubbled with N_2_ for 5 min. Further, balloons inflated with N_2_ were attached to each tube to generate an inert, oxygen-free atmosphere. After that, all tubes were placed in a thermostatic bath at 70 °C for 24 h. Once the synthesis was completed, the MIPs were removed from the tubes, sieved, ground into powder, and washed with 300 mL of ethanol followed by 200 mL of ultrapure water. Finally, the polymer was dried in an oven at 40 °C. The non-molecularly imprinted polymers (NIPs) were synthesised under the same conditions and following the same protocol as MIPs, but in the absence of the imprinting molecules.

#### 2.2.2. Removal of the Imprinting Molecules

The removal of the imprinting molecules (BPA or PG) was performed with dry MIPs. They were placed in 250-mL beakers. To each beaker, 150 mL of methanol and 50 mL of acetic acid was added. The mixture was stirred at 50 rpm for 15 min and incubated for 1 h at ambient temperature. After incubation, the supernatant was removed by decantation. The procedure was repeated five times. The final washing was performed with an additional 50 mL of methanol. After that, polymers were filtrated, and dried on Petri dishes in a vacuum oven at 40 °C.

#### 2.2.3. Grinding and Sieving of MIPs and NIPs

After grinding, MIPs were sieved and the fraction of particles between 180 μm and 250 μm was used in all further studies.

#### 2.2.4. Characterisation of MIPs and NIPs Using Infrared Spectroscopy (FT-IR) and Electron Scanning Microscope (SEM)

The FT-IR spectra of the MIPs samples in KBr pellets were obtained on a Nicolet Magna 550 (Madison, WI, USA), in the range of 4000–400 cm^−1^. Additionally, the samples were analysed by SEM in JEOL JSM-6380 LV (Tokyo, Japan) using an acceleration voltage of 20 kV and different magnification ranges. The coating with gold was fabricated under vacuum in an sputter coater SPI 11427-AX (West Chester, PA, USA), for 30 s.

#### 2.2.5. Detection of BPA and PG Using High Performance Liquid Chromatography (HPLC)

Calibration standards of BPA and PG were prepared in 250-mL volumetric flasks in acetonitrile (BPA) and methanol (PG). Concentrations of the stock solutions were 200 mg/L for BPA and 8.8 mg/L for PG. From those stock solutions, suitable dilutions were made and used later for the calibration curves. The calibration curve for BPA was obtained between 1 mg/L and 5 mg/L and for PG between 0.88 mg/L and 8.8 mg/L. Solvents selected for the separation processes were ethanol, water, and acetonitrile. They were filtered, sonicated, and kept for further use.

The detection of BPA was performed using HPLC, YL 9100 system with quaternary pump YL9110, Degasser YL9101, Fluorescence Detector model G1321A with autosampler YL9150, integration system, and data logging Clarity-Chromatography SW. The detection of PG was performed using HPLC, Merck-Hitachi (Tokyo, Japan) with UV detector L7420 (Merck-Hitachi, Tokyo, Japan). The chromatographic conditions of the analysis of BPA and PG are listed in Table 2.

#### 2.2.6. Retention Studies for MIPs and NIPs in Batch Method

To 15-mL falcon tubes, 50 mg of MIP or NIP was added, followed by 5 mL of standard solution of BPA (3.12 mg/L) or PG (5.28 mg/L). This mixture was stirred for 1 h on a reciprocal shaker and centrifuged for 5 min. The supernatant was filtered through a polyvinyldene fluoride (PVDF) membrane filter (0.22 μm) and placed into Eppendorf tubes. The concentration of BPA and PG in the supernatant was measured by HPLC with fluorescence or UV detectors.

#### 2.2.7. Kinetics Studies

In order to measure the retention capacity at different contact times, 50 mg of MIP or NIP and 5 mL of standard solution of BPA (3.12 mg/L) or PG (5.28 mg/L) were added to 10 falcon tubes. The tubes were shacked on reciprocal shaker for 0, 5, 10, 15, 30, 45, 60, 90, 120, and 150 min. After that, they were quickly transferred and centrifuged for 3 min at 4000 rpm. The supernatant was filtered with a PVDF membrane filter (0.22 μm) and placed into Eppendorf tubes. The concentration of BPA in the supernatant was measured by HPLC with a fluorescence detector.

#### 2.2.8. Adsorption Isotherms

The adsorption isotherms were performed using eight different concentrations of BPA in the range of 25 to 200 mg/L and four different temperatures (25, 30, 35, and 40 °C), and eight different concentrations of PG in the range of 0.8 to 88 mg/L. To each falcon tube, 50 mg of MIP or NIP was added, followed by 5 mL of the standard solution of BPA or PG at different concentrations. After that they were sonicated for 24 h at 140 rpm at ambient temperature and centrifuged for 2 min at 4000 rpm. Supernatants were transferred into Eppendorf tubes and the concentrations of BPA or PG were subsequently measured by HPLC with a fluorescence detector for BPA and a UV-visible detector for PG.

#### 2.2.9. Cross-Reactivity of the MIPs in Real Water Samples

The cross-reactivity of the MIPs was tested in drinkable water samples by using four commonly used organic compounds (diosgenin, diphenilamyne, PG and BPA) in two different concentrations, depending on the method of detection.

#### 2.2.10. Reusability Capacity of MIPs

First, 50 mg of MIP 2 was loaded with 5 mL of standard solution of BPA (3.12 mg/L) or PG (5.18 mg/L). After the analyte was adsorbed on the MIPs, the concentration of the residue in the supernatant was calculated. This allowed the estimation of how much analyte was retained in the MIP. Furthermore, the percentage of the liberation of the analyte was calculated based on the previous knowledge of its amount adsorbed into the MIP and elution using four different proportions of eluents: methanol/acetonitrile (65/35 *v*/*v*), acetonitrile, ethanol/methanol (30/70 *v*/*v*), and methanol.

## 3. Results

### 3.1. Synthesis and Characterisation of MIPs and NIPs

**Figure 1:**
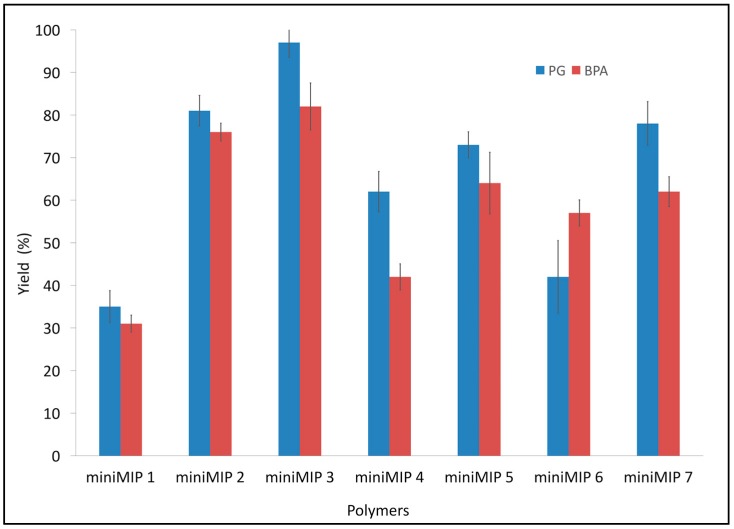
Yield of the synthesis of miniMIPs in different experimental conditions. Error bars represent ±1 standard deviation. All experiments were performed in triplicate.

Following the experimental design, seven MIPs for PG and seven MIPs for BPA (so-called miniMIPs) were produced. They were prepared in the small quantities in order to optimise conditions of the final polymerisation and the synthesis of suitable polymers that could be used in all further experiments. After initial optimisation, two miniMIPs with the best performance were selected for each analyte and prepared in larger quantities. Subsequently, their names changed into MIPs/NIPs. Figure 1 shows the yield for each miniMIP synthesised with acrylic acid as a functional monomer and BPA and PG as templates.

The above bar chart shows that in both cases (BPA or PG) miniMIP 3 had the highest yield, 96.6% and 73.1% for PG and BPA, respectively. It can also be seen that miniMIP 1 had the lowest yield in both cases, PG 35.1% and 33.3% BPA. This correlation between the yield of miniMIP 3 and miniMIP 1 can be attributed to the experimental conditions of the synthesis (see Table 1). miniMIP 3 had a maximum quantity of cross-linker and a minimum quantity of solvent. Those conditions might lead to the presence of a gel effect and therefore a not well homogenised mixture, which increased the viscosity of the miniMIP and amplified the polymerisation reaction rate. As a consequence, the radical reaction might be chaotic, resulting in a slower rate of termination. The monomer-template-solvent interactions under those conditions could form a highly cross-linked random copolymer.

**Figure 2:**
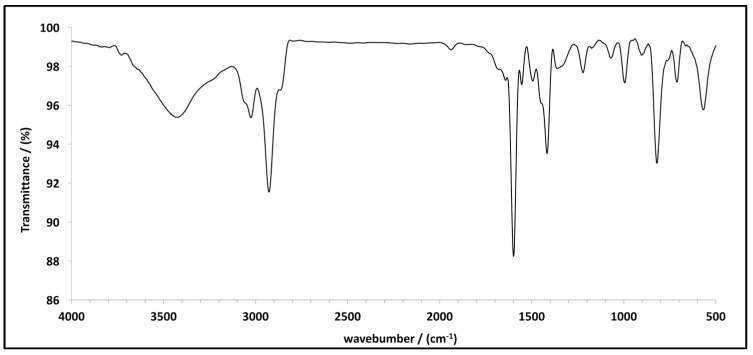
FT-IR spectrum of miniMIP 3 prepared for BPA.

Figure 2 shows the FT-IR spectra of miniMIP 3 based on acrylic acid for BPA. The spectra represent typical absorption bands associated with the functional groups of the monomer in miniMIP 3. One of them is placed between 3500 and 3400 cm^−1^. This band corresponds to the stretching vibrations of hydroxyl groups (-OH) in the acrylic acid. Between 3000 and 2900 cm^−1^, a peack can be seen which was attributed to the signal from carbon and hydrogen (corresponding to the benzene ring 1,4-divinylbenzene). Another strong absorption band can be seen around 1600 cm^−1^, associated with the tension of carbon and oxygen (C=O) from the acrylic acid. C-H wags of benzene rings can be seen at 801 cm^−1^.

**Figure 3:**
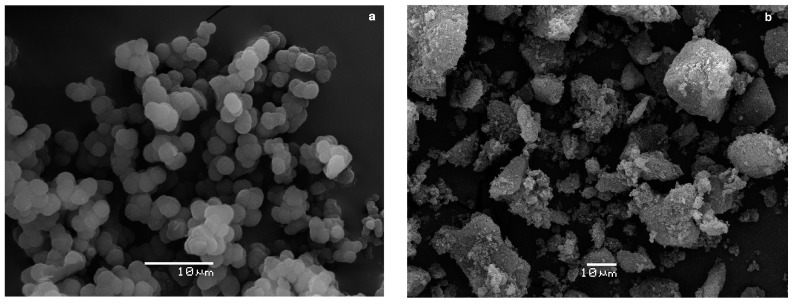
SEM microscopies of (**a**) miniMIP 1; (**b**) miniMIP 3 for BPA.

The morphologies of miniMIP 1 and miniMIP 3 were analysed by electron scanning microscopy (SEM). The results are shown in Figure 3a,b for miniMIP 1 and miniMIP 3, respectively. As we can see, there are significant differences in the general morphology, size, and shape of the produced miniMIPs. These differences can be related to the previous observation and the differences in the amount of cross-linker and solvent during the syntheses.

miniMIP 1 (see Figure 3a) was polymerised with 10 mL of solvent. The final product took the form of powder and pellets, and the grains were more homogenised than those of miniMIP 3. The lower amount of the solvent might cause rigid structure-shaped cuts, which could be formed in the grinding process of miniMIP 3 (see Figure 3b).

### 3.2. High Performance Liquid Chromatography (HPLC)

An analytical method that allowed the identification and quantification of PG and BPA was developed using chromatographic standards. The analysis was performed in water using HPLC with a fluorescence detector for BPA and a UV-Vis detector for PG. The conditions are summarised in Table 2. A characteristic peak for PG can be seen at 16 min of the measurement and the peak for BPA can be seen at 12 min. The other signals correspond to the solvent signal and the dead time signal (see Supplementary Materials).

The quantification of both analytes was made based on calibration curves. To obtain the analytical parameters of the calibration curve, 20 different blanks were measured. The limit of detection (LOD) was obtained by calculating three times the standard deviation of the blanks divided by the slope of the curve. The limit of quantification (LOQ) was calculated to be three times the LOD [45,46]. To determine the calibration curves, all concentrations were measured in triplicate.

The calibration curve for BPA showed an LOD of 0.015 µg/mL and an LOQ of 0.045 µg/mL. The calibration curve for PG showed an LOD of 11.3 µg/mL and an LOQ of 34.0 µg/mL.

### 3.3. BPA and PG Retention Capacity of the Synthesised miniMIPs

**Figure 4:**
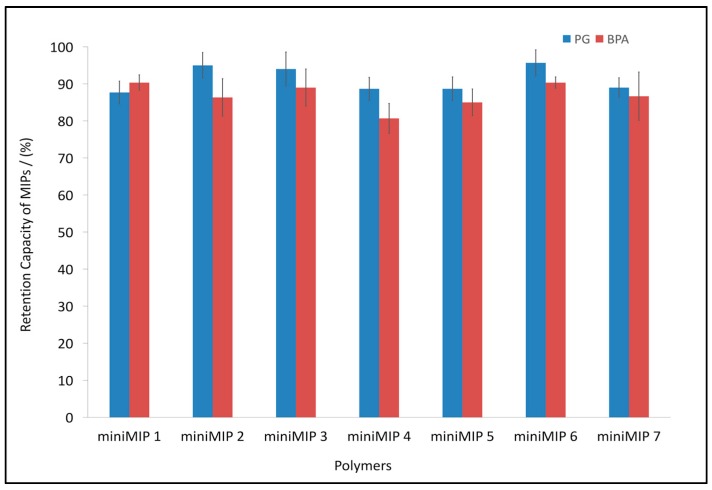
PG and BPA retention capacities of miniMIPs; an initial concentration of PG (5.28 mg/L), initial concentration of BPA (3.12 mg/L), and 50 mg of miniMIPs synthesised according to the experimental design. Error bars represent ±1 standard deviation. Experiments were performed in triplicate.

Figure 4 shows the retention capacities for each miniMIP. In both cases (BPA and PG), the retention capacity was about 100%. Unfortunately, it was not possible to find a tendency between retention capacities of each miniMIP and different amounts of solvent, cross-linker, and template. Therefore, an explanation for the high retention could be that different types of interactions between the template and the functional monomer played an important role (the formation of hydrogen bonds between carboxylic acid groups in acrylic acid and carbonyl groups in the case of PG or alcohol groups for BPA) [47].

In our previous work [17], it was demonstrated that one variable (cross-linker) was the most important parameter during the synthesis of miniMIPs. In order to test that statement, the yield and retention capacity of miniMIPs were analysed using different amounts of cross-linker. The results showed a tendency similar to that of the previous findings. While the amount of cross-linker increased, the yield of synthesised miniMIPs increased. In the presence of a lower amount of cross-linker, the yield decreased. Based on those results, miniMIPs with the best performance (highest yield and highest retention) could finally be chosen. Therefore, miniMIP 2 and miniMIP 3 were selected as the most efficient polymers. As mentioned earlier, those MIPs and their NIP controls were then synthesised again but in larger quantities, and they were renamed as MIP 2 and MIP 3, respectively. The yields of the polymers were 74.2% (BPA) and 83.6% (PG) for MIP2; 79.6% and 87.1 for the control NIP 2; 87.2% (BPA) and 92.4% (PG) for MIP 3; and 81.4% and 91.1% for the control NIP 3.

### 3.4. BPA and PG Retention Studies by Selected MIPs and NIPs

**Figure 5:**
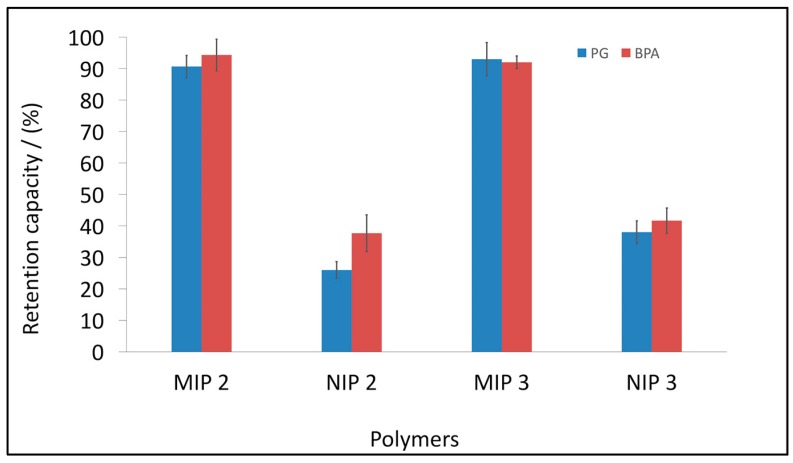
Retention of PG and BPA for two selected MIPs and NIPs. The concentration of PG was 5.28 mg/L and the concentration of BPA was 3.12 mg/L. Error bars represent ±1 standard deviation. Experiments were performed in triplicate.

The retention capacities of the selected MIPs are presented in Figure 5. During the analysis, 50 mg of MIP or NIP was added to 15-mL falcon tubes, followed by 5 mL of standard solution of BPA (3.12 mg/L) or PG (5.28 mg/L).

In Figure 5, a significant difference in the retention capacity between MIPs and NIPs can be observed, which suggests the high efficiency of the imprinting process. MIP 2 for PG shows a retention rate 3.5 times higher than that of the control NIP 2, and 2.5 times higher in the case of BPA. Similar trend can be seen using MIP 3. The difference in the retention rate between MIP 3 and NIP 3 is 2.44 times higher for PG and 2.24 times higher for BPA.

### 3.5. Adsorption Isotherms

Adsorption isotherms were made with MIP 2, MIP 3, NIP 2, and NIP 3 in order to determine the maximum adsorption capacity of the polymers (see Supplementary Materials). To analyse the experimental data for adsorption isotherms, Langmuir and Freundlich models were applied. Fitting adsorption data helped to accurately describe the experimental results and to find the most appropriate model for the analysed MIPs and NIPs. Table 3 and Table 4 show the parameters obtained for each linearised isotherm model for MIPs made for BPA, and Table 5 and Table 6 show the parameters obtained for each linearised isotherm model for the MIPs made for PG. Both models (Freundlich and Langmuir) showed excellent linearity (R^2^ ≥ 96%). From the linearised Freundlich isotherm, we obtained the intensity parameter “n” and adsorption affinity of the polymer “K” (see Table 3). The results showed a significant difference in the affinity (K) between MIP 3 and NIP 3 at 308 K. MIP 3 exhibited a value of “K” 2.7 times higher than that of the control NIP 3. This could indicate the better affinity of the MIP than the NIP because of the presence of a specific cavity for the recognition of BPA. Data from the linearised Langmuir isotherms are shown in Table 4. From the linearised Langmuir isotherm, we obtained the maximum adsorption capacity (Qmax) of the MIPs and NIPs. The highest value of Qmax for BPA was observed for MIP 2 at 308 K (19.53 mg/g) while the Qmax of the control (NIP 2) was 1.876 mg/g. Therefore, MIP 2 retained BPA 9.88 times more than NIP 2. For MIP 3, the highest value of Qmax was observed at 303 K (19.46 mg/g), while the Qmax of the control (NIP 3) was 2.584 mg/g. Hence, MIP 3 retained 7.53 times more BPA than NIP 3. In Table 6, the results from the linearised isotherm for PG can be seen. The highest value of Qmax was observed in MIP 2 at 308 K with value of 21.49 mg/g, while the Qmax for the control (NIP 2) was 2.593 mg/g. Thus, MIP 2 retained PG 8.28 times more than the control. Consequently, the highest value of Qmax for MIP 3 was observed at 303 K 17.63 mg/g, while the Qmax for the control (NIP 3) was 1.951 mg/g. MIP 3 retained PG 9.03 times more than NIP 3.

### 3.6. Kinetic Study of the Retention of BPA and PG in MIPs

**Figure 6:**
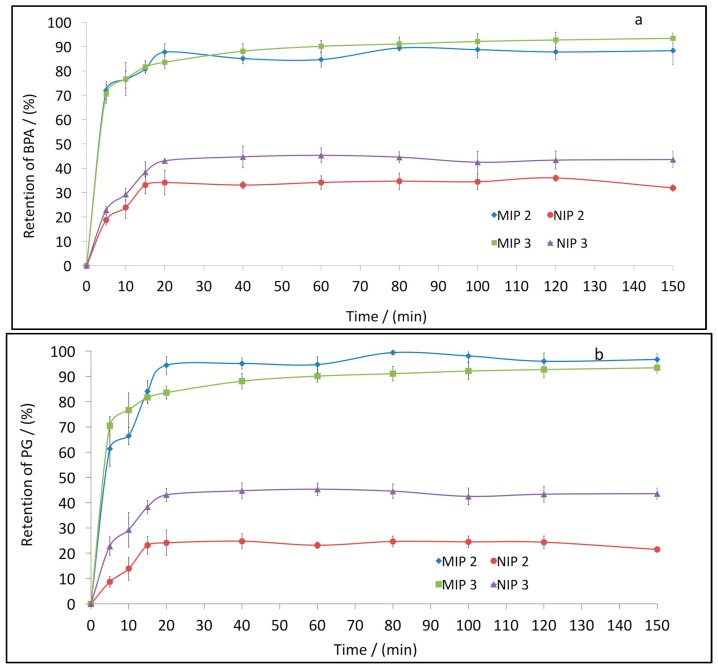
Kinetic study of the retention of BPA (**a**) and PG (**b**) with MIPs and NIPs. Error bars represent ±1 standard deviation. Experiments were performed in triplicate.

Figure 6 shows the adsorption of BPA. It can be seen that the adsorption reached the equilibrium phase after a very short period of time (less than 20 min). This could indicate the high specific binding of the imprinted polymers. Additionally, the percentage of the retention capacity was significantly higher for MIPs than for NIPs.

### 3.7. Cross-Reactivity of the MIPs in Water Samples

The cross-reactivity of MIPs prepared for PG (MIPs-PG) and BPA (MIPs-BPA) was tested against structurally similar compounds and other endocrine disruptors rather than target molecules. To test the cross-reactivity of MIPs-PG, diosgenin was used as a structural analogue and BPA was used as an endocrine disruptor. In the case of MIPs-BPA, diphenylamine was used as a structurally similar compound and PG was used as an endocrine disruptor. All experiments were performed in triplicate. The cross-reactivity of MIPs was analysed in ultrapure water against one analyte at a time, at two different concentrations (1 mg/L and 3 mg/L). In all cases, MIPs showed no significant cross-reactivity with a rather high recovery of the tested analytes, more than 85%. The lowest cross-reactivity was observed for MIPs-PG tested against BPA (with a recovery of 88.2 ± 2.3%) and MIPs-BPA tested against PG (87.2 ± 5.7%). Additionally, the slightly lower recovery of 85.5 ± 4.2% (MIPs-PG against diosgenin) and 82.9 ± 3.5% (MIPs-BPA against diphenylamine) suggested a higher cross-reactivity in the presence of structural analogue molecules. This certain degree of cross-reactivity can be related to the fact that MIPs do not generally show absolute specificity for target molecules and can also exhibit certain interactions with other structurally related molecules or cross-reactants with a similar distribution of functional groups [47].

### 3.8. Evaluation of the Reusability Capacity of MIPs

The reusability capacity of a sorbent is one of the most critical properties that must be tested before a new material can be applied in microextraction [48]. For this reason, the reusability of MIP 1 and MIP 3 was analysed in order to check their potential for future application in SPE.

**Figure 7:**
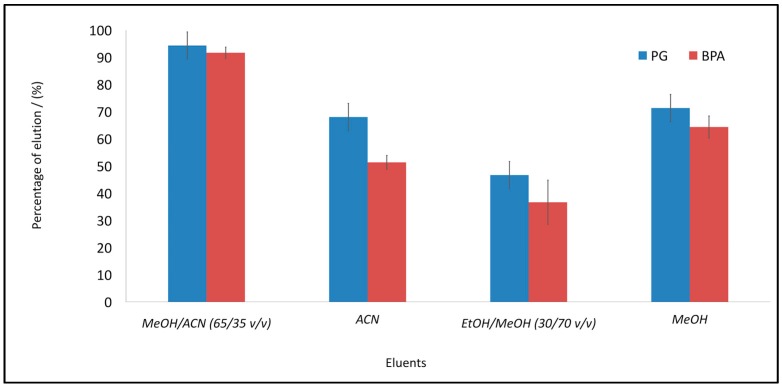
Elution of PG and BPA from MIP 2, with four different solvents. Error bars represent ±1 standard deviation. Experiments were performed in triplicate.

First, 50 mg of MIP 2 was loaded with 5 mL of standard solution of BPA (3.12 mg/L) or PG (5.18 mg/L). After the analyte was adsorbed on the MIPs, the concentration of the remanent analyte in the supernatant was calculated and the amount of the analyte retained in the MIPs was estimated. Knowing the amount adsorbed into the MIP, the percentage of the liberation of the analyte was calculated using four different proportions of eluents (See Figure 7).

When using the batch method, four possible eluents were applied: acetonitrile, methanol, methanol/acetonitrile (65:35 *v*/*v*), and ethanol/methanol (30:70 *v*/*v*). It was found that the best elution process employed the methanol/acetonitrile mixture, with over 93% elution efficiency (see Figure 7).

**Figure 8:**
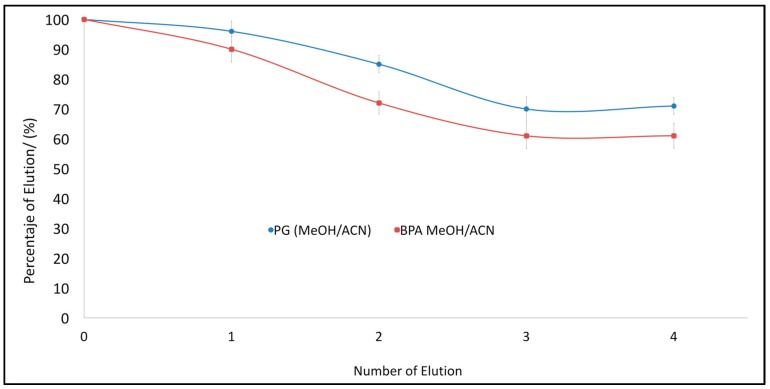
Elution capacity of PG and BPA from consecutive loading and unloading cycles in MIP 2. Error bars represent ±1 standard deviation. Experiments were performed in triplicate.

After determining the most suitable mixture of the solvents for the elution of analytes adsorbed in MIPs, the analysis of the reuse of the MIP was continued (See Figure 8). A series of tests of adsorption and desorption of PG and BPA onto MIP 2 was performed in order to evaluate how many consecutive experiments can be carried out using one MIP.

### 3.9. Evaluation of the Adsorption Capacity in Real Water Samples

MIP 2 was used to evaluate its adsorption capacity for BPA and PG in real environmental water samples (tap and bottled water). Each sample of water was spiked with PG (5.28 mg/L) and BPA (3.12 mg/L). The experimental conditions and further analysis were the same as those described in Section 3.4. Therefore, to a 15-mL falcon tube, 50 mg of MIP 2 was added, followed by 5 mL of spiked water sample. This mixture was stirred for 1 h and centrifuged for 5 min. The supernatant was filtered and placed in an Eppendorf tube. The concentration of BPA and PG in the supernatant was measured by HPLC with fluorescence or UV detectors.

For all samples, the retention capacity was higher than 87%. In the case of BPA, it was 87.2% (±2.3%) and 89.8% (±2.1%) for tap and bottled water, respectively. In the case of PG, the results were similar, 91.2% (±2.3%) and 89.7% (±3.1%) for tap and bottled water, respectively. All measurements were performed in triplicate. In comparison with ultrapure water (see Figure 5), the final results demonstrated excellent performance and potential application in the analysis of real complex samples.

## 4. Conclusions

Using radical polymerisation, it was possible to obtain high yields of different polymers (up to 96.6%). The optimal conditions for the synthesis of MIPs for BPA and PG were defined following experimental design, which helped to eliminate all insignificant factors for the polymerisation and therefore reduced the large number of syntheses.

The limits of detection and quantification of the analytes in water were obtained using HPLC with a fluorescence detector for BPA and a UV-visible detector for PG. For BPA, the LOD was 0.015 mg/mL and the LOQ was 0.045 mg/mL. For PG, the LOD was 11.3 mg/mL and the LOQ was 34.0 mg/mL.

The kinetic studies showed rapid BPA and PG retention, in less than 15 min of contact. The adsorption isotherms were analysed using Langmuir and Freundlich models. Results indicated that each cavity allowed binding only a single target molecule, which created a monolayer and later a multilayer on the surface of the polymer.

Overall, significant differences between retention capacities for MIPs and NIPs were observed. The Qmax of the retention of BPA for MIP 2 was 9.88 times higher than that for the control (NIP 2), and for MIP 3 was it 7.53 times higher than that for the control (NIP 3). The Qmax of the retention for PG for MIP 2 was 8.28 times higher than that for the control (NIP 2), and for MIP 3 it was 9.03 times higher than that for the control (NIP 3). Additionally, there was no significant cross-reactivity reported between MIPs and other related molecules. When the MIPs were applied in real water samples the results were practically identical to those of ultrapure water. This proved the great efficiency of the proposed materials and their potential application in environmental samples. Therefore, further work should include the testing of different mobile phases for the batch experiments, e.g., by decreasing the polarity of a media we could try to decrease the non-specific interactions between analytes and the active sites of imprinted polymers.

## Figures and Tables

**Table 1:** Experimental conditions for the synthesis of molecularly imprinted polymers (MIPs) according to the experimental design.

Experiment	Cross-Linker (DVB)/mol % of Monomer	Solvent (Acetonitrile) mL	Template (BPA or PG)/mol % of Monomer
miniMIP1	50	10	10
miniMIP2	50	2	30
miniMIP3	300	2	10
miniMIP4	300	10	30
miniMIP5	175	6	20
miniMIP6	175	6	20
miniMIP7	175	6	20

**Table 2:** Chromatographic conditions for the analysis of bisphenol A and progesterone.

Parameters	Analysis of BPA	Analysis of PG
Mobile phase	H_2_O:Acetonitrile (55:45)	Methanol:H_2_O (70:30)
Column	Lichrospher RP-C8 Symmetry^®^ column, 5 μm, 4.6 × 250 mm	Lichrospher RP-C8 Symmetry^®^ column, 5 μm, 4.6 × 250 mm
Mobile phase flow (mL/min)	1	1
Column temperature (°C)	25	25
Time of analysis (min)	5	15
Injection volume (µL)	10	50
Detector	Fluorescence	UV-Vis
Wavelength excitation/emission (nm)	230/315	248

**Table 3:** Parameters of the Freundlich linearisation isotherms for the retention of BPA with MIPs and NIPs.

	298 K		303 K		308 K		313 K	
Polymer	n	K (L/mg)	n	K (L/mg)	n	K (L/mg)	n	K (L/mg)
MIP 2	0.851	3.336	0.589	0.867	1.369	11.32	1.850	5.235
NIP 2	0.780	2.940	0.661	0.579	0.876	2.677	0.851	0.620
MIP 3	1.340	50.15	0.607	35.03	1.620	80.33	1.254	76.51
NIP 3	0.490	29.02	0.540	28.67	0.473	29.85	0.320	26.42

**Table 4:** Parameters of the Langmuir linearisation isotherms for the retention of BPA with MIPs and NIPs.

	298 K		303 K		308 K		313 K	
Polymer	Qmax (mg/g)	b (L/mg)	Qmax (mg/g)	b (L/mg)	Qmax (mg/g)	b (L/mg)	Qmax (mg/g)	b (L/mg)
MIP 2	14.06	0.230	13.61	0.056	19.53	1.321	1.850	5.235
NIP 2	2.49	0.076	4.02	0.096	1.876	2.677	0.851	0.620
MIP 3	12.85	1.36	19.46	0.481	17.62	80.33	1.254	76.51
NIP 3	2.501	0.020	2.584	0.018	2.473	29.85	0.320	24.42

**Table 5:** Parameters of the Freundlich linearisation isotherms for the retention of PG with MIPs and NIPs.

	298 K		303 K		308 K		313 K	
Polymer	n	K (L/mg)	n	K (L/mg)	n	K (L/mg)	n	K (L/mg)
MIP 2	0.636	2.167	0.462	1.046	1.618	10.72	2.010	4.351
NIP 2	0.408	1.245	0.322	0.351	0.762	3.485	0.895	1.358
MIP 3	1.070	20.24	0.701	3.423	1.981	28.13	1.387	21.69
NIP 3	0.355	17.43	0.431	2.589	0.553	3.618	0.391	4.071

**Table 6:** Parameters of the Langmuir linearisation isotherms for the retention of PG with MIPs and NIPs.

	298 K		303 K		308 K		313 K	
Polymer	Qmax (mg/g)	b (L/mg)	Qmax (mg/g)	b (L/mg)	Qmax (mg/g)	b (L/mg)	Qmax (mg/g)	b (L/mg)
MIP 2	12.18	0.184	16.17	0.174	21.49	1.047	6.850	17.35
NIP 2	1.75	0.103	3.29	0.052	2.593	0.869	1.101	1.940
MIP 3	10.19	0.272	18.62	0.218	17.63	15.84	2.061	31.25
NIP 3	2.428	0.015	3.105	0.085	1.951	1.89	0.269	9.71

## Supplementary Materials

The following supplementary materials are available online at http://www.mdpi.com/2073-4360/10/6/679/s1.
