# Supplementary material for: Molecularly Imprinted Polymers for the Selective Extraction of Bisphenol A and Progesterone from Aqueous Media
Source: Polymers (Basel). 2018 Jun 19;10(6):679. doi: 10.3390/polym10060679 (PMC6404127; doi:10.3390/polym10060679)
Supplement: Supplementary file 1 [file polymers-10-00679-s001.pdf]

Suplementary materials

Molecularly Imprinted Polymers for the selective extraction of Bisphenol A and Progesterone from aqueous media

César Cáceres[1], Catalina Bravo[2], Bernabé Rivas[1], Ewa Moczko[3], Pedro Sáez[4] ,Yadiris Garcia, Eduardo Pereira[2]*

S1. Study of different monomers -Acrylic Acid, 4-Vinylpyridine and Acrylamide, in the table 1, 2 and 3 it can be seen the experimental design for the synthesis of all the MIPs.

S1. **Table 1**, experimental conditions for the synthesis of MIPs for Acrylic Acid

Experiment	Cross-linker (DVB) /mol % of monomer	Solvent (Acetonitrile) /mol % of monomer	Template (BPA or PG) /mol % of monomer
miniMIP1	50	10	10
miniMIP2	50	2	30
miniMIP3	300	2	10
miniMIP4	300	10	30
miniMIP5	175	6	20
miniMIP6	175	6	20
miniMIP7	175	6	20

S1. **Table 2**, experimental conditions for the synthesis of MIPs for 4-VPy

Experiment	Cross-linker (DVB) /mol % of monomer	Solvent (Acetonitrile) /mol % of monomer	Template (BPA or PG) /mol % of monomer
miniMIP8	50	10	10
miniMIP9	50	2	30
miniMIP10	300	2	10
miniMIP11	300	10	30
miniMIP12	175	6	20
miniMIP13	175	6	20
miniMIP14	175	6	20

S1. Table 3, experimental conditions for the shyntesis of MIPs for Acrilamide

Experiment	Cross-linker (DVB) /mol % of monomer	Solvent (Acetonitrile) /mol % of monomer	Template (BPA or PG) /mol % of monomer
miniMIP15	50	10	10
miniMIP16	50	2	30
miniMIP17	300	2	10
miniMIP18	300	10	30
miniMIP19	175	6	20
miniMIP20	175	6	20
miniMIP21	175	6	20

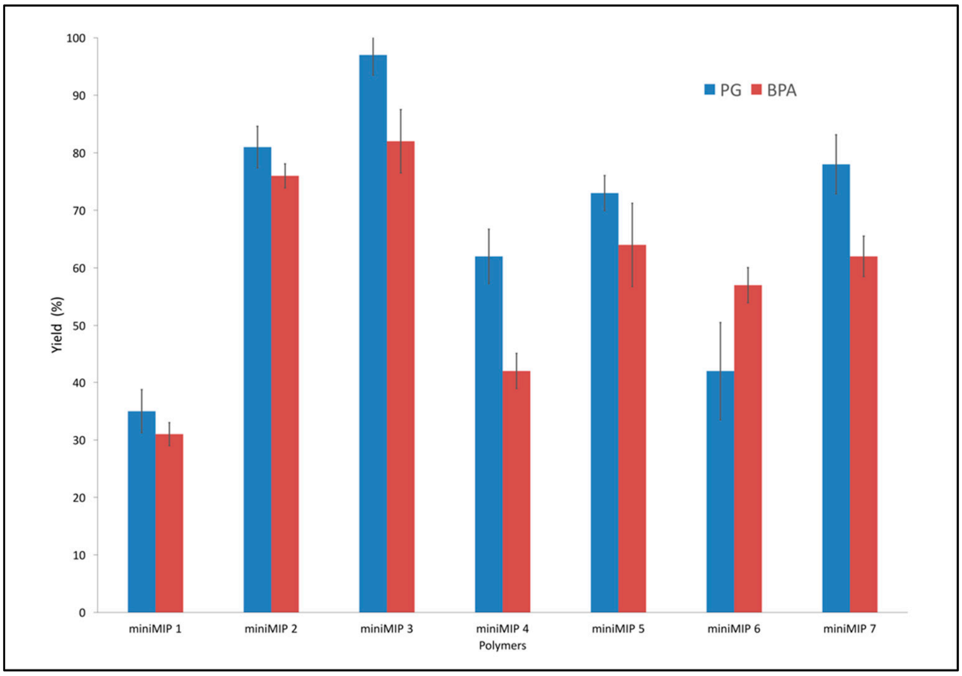

S1, **Figure 1.** Yield of the synthesis of miniMIPs in different experimental conditions (monomer; Acrilic Acid). Error bars represent ±1 standard deviation. All experiments were performed by triplicate.

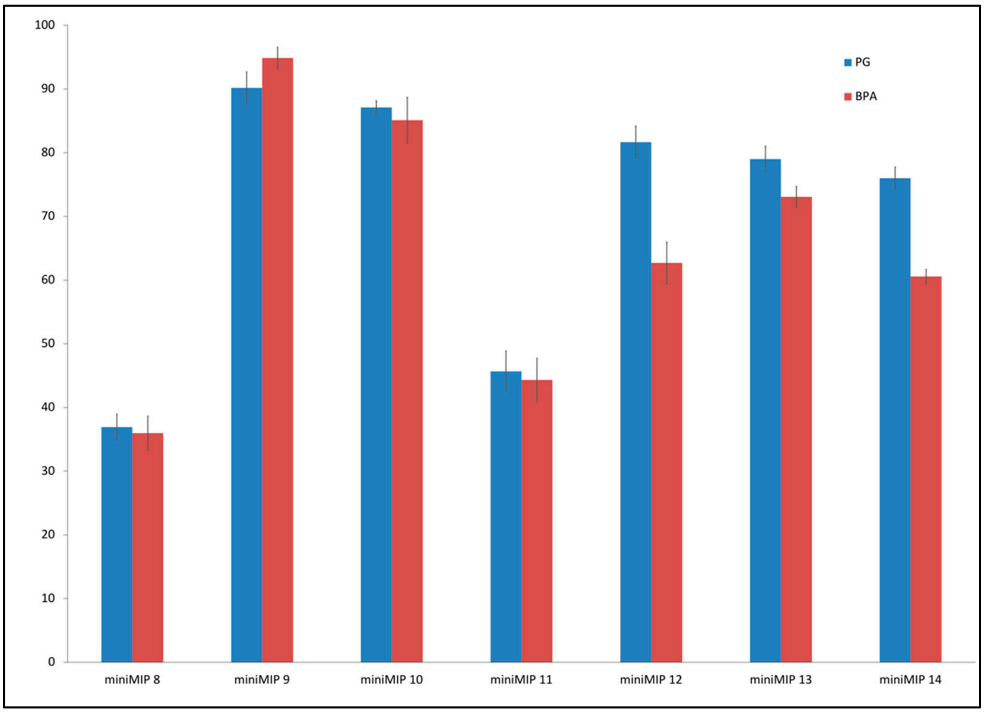

S1, **Figure 2.** Yield of the synthesis of miniMIPs in different experimental conditions (monomer; 4-Vpy). Error bars represent ±1 standard deviation. All experiments were performed in triplicates.

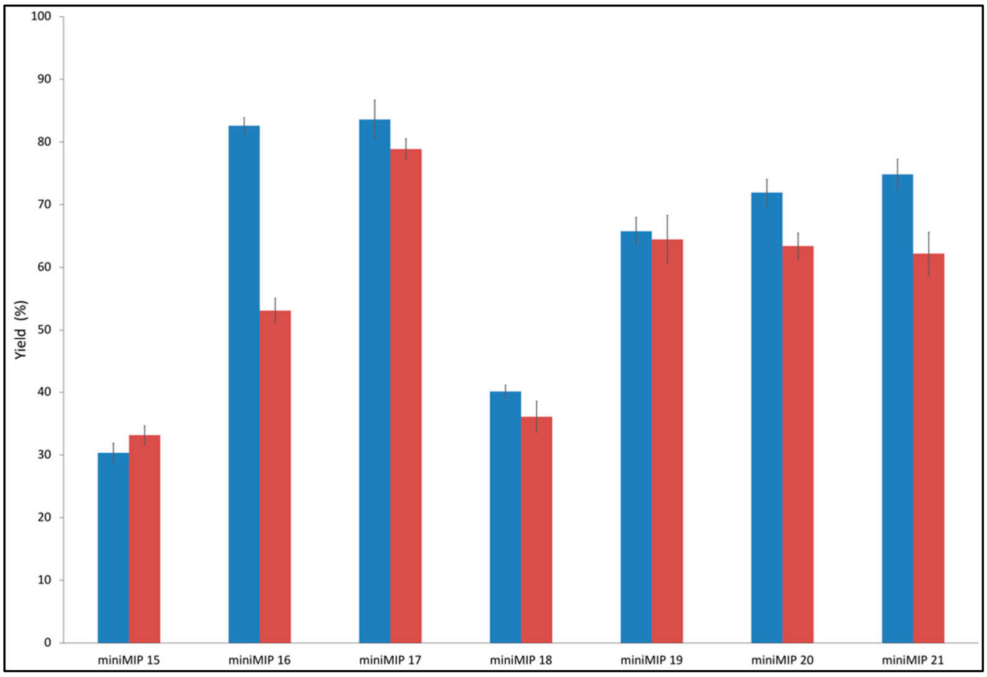

S1, **Figure 3.** Yield of the synthesis of miniMIPs in different experimental conditions (monomer; Acrilamide). Error bars represent ±1 standard deviation. All experiments were performed in triplicates.

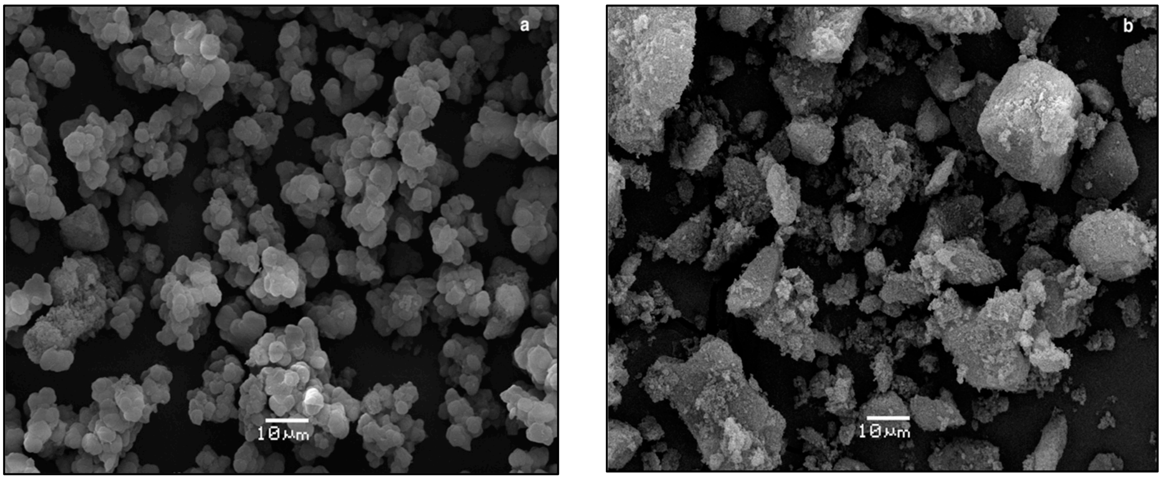

S1, **Figure 4.** SEM microscopies of (a) miniMIP1, (b) miniMIP3 for BPA. Monomer AA.

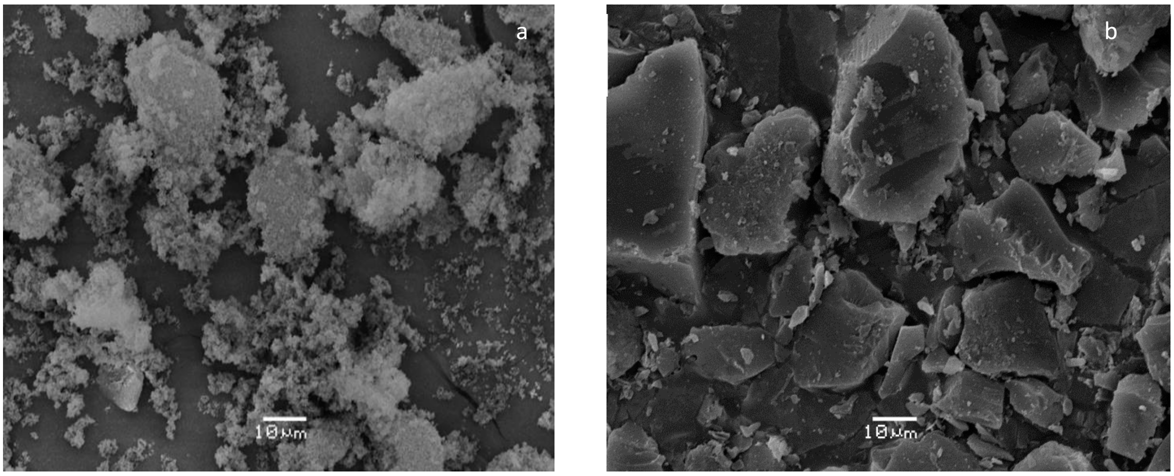

S1, **Figure 5.** SEM microscopies of (a) miniMIP8, (b) miniMIP10 for BPA. Monomer 4-Vpy

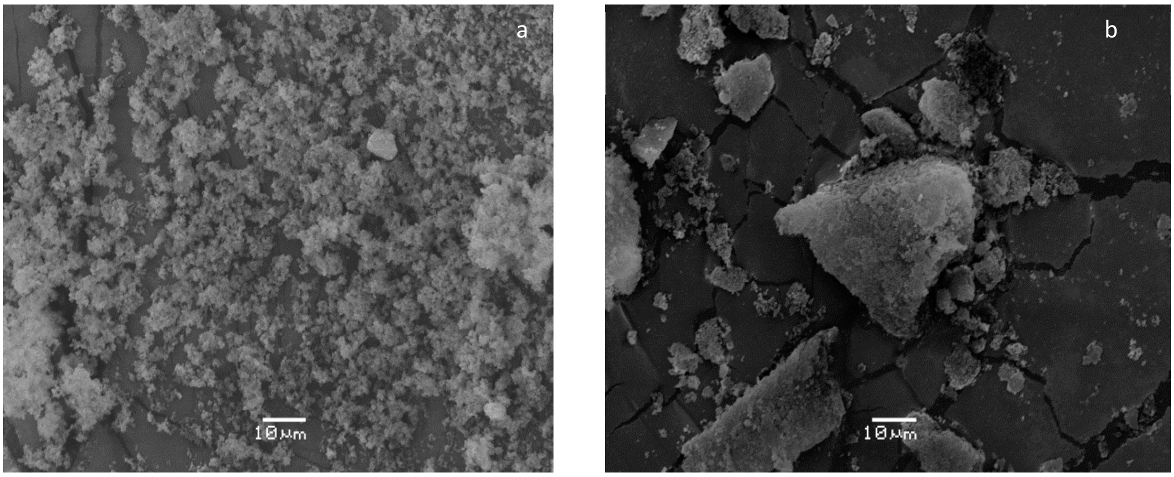

S1, **Figure 6.** SEM microscopies of (a) miniMIP15, (b) miniMIP17 for BPA. Monomer AAm

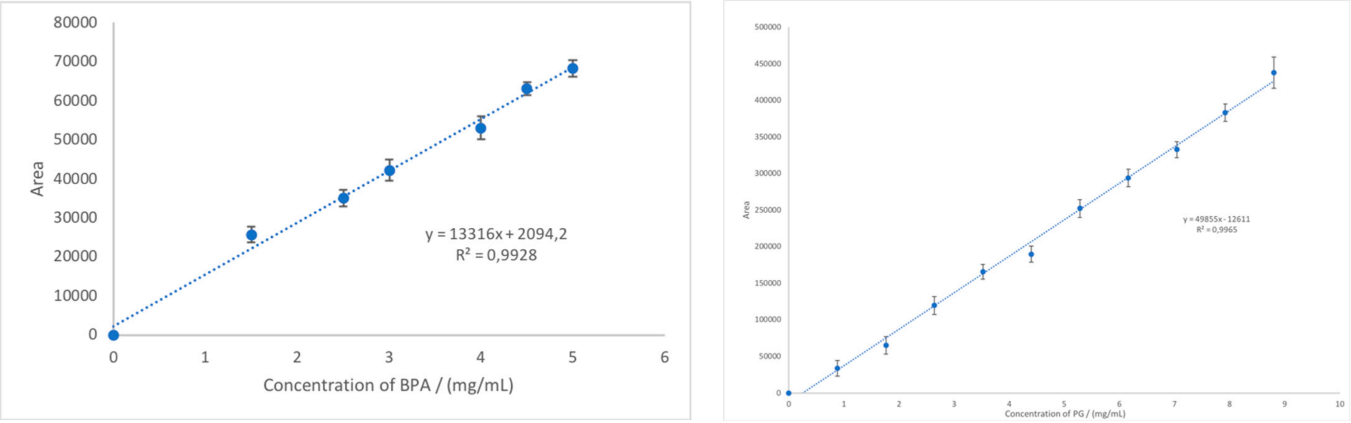

S1, **Figure 7.** Calibration curves of BPA and PG, experimental conditions are in the manuscript.

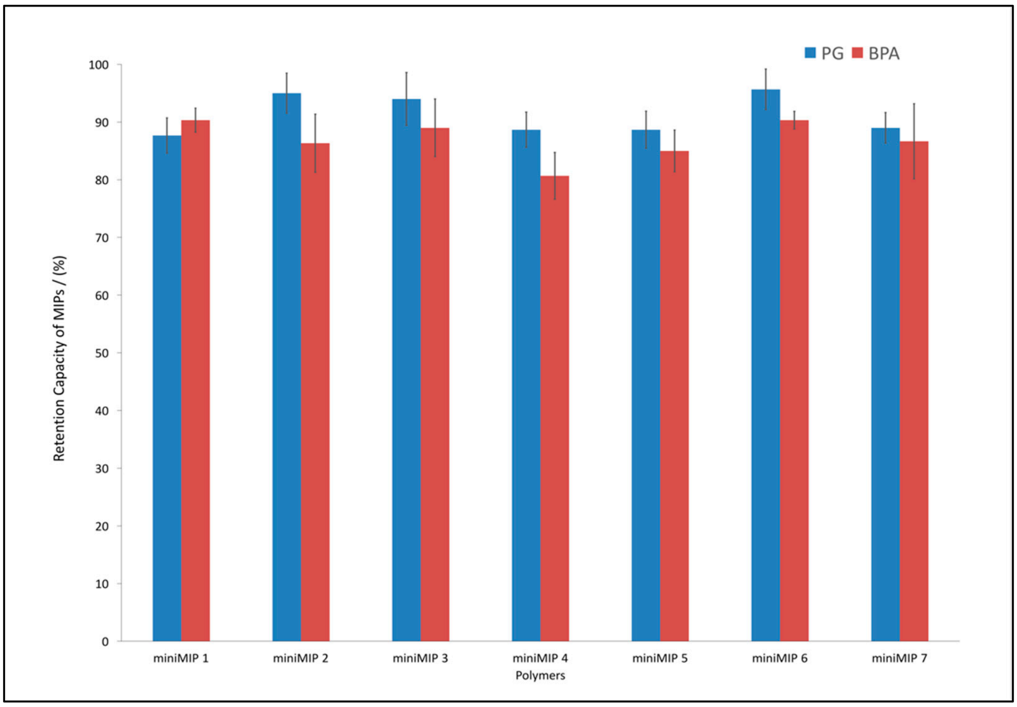

S1. **Figure 8.** PG and BPA retention capacities of miniMIPs made with AA like monomer, synthesized according to the experimental design. Error bars represent ±1 standard deviation. Experiments were performed by triplicate.

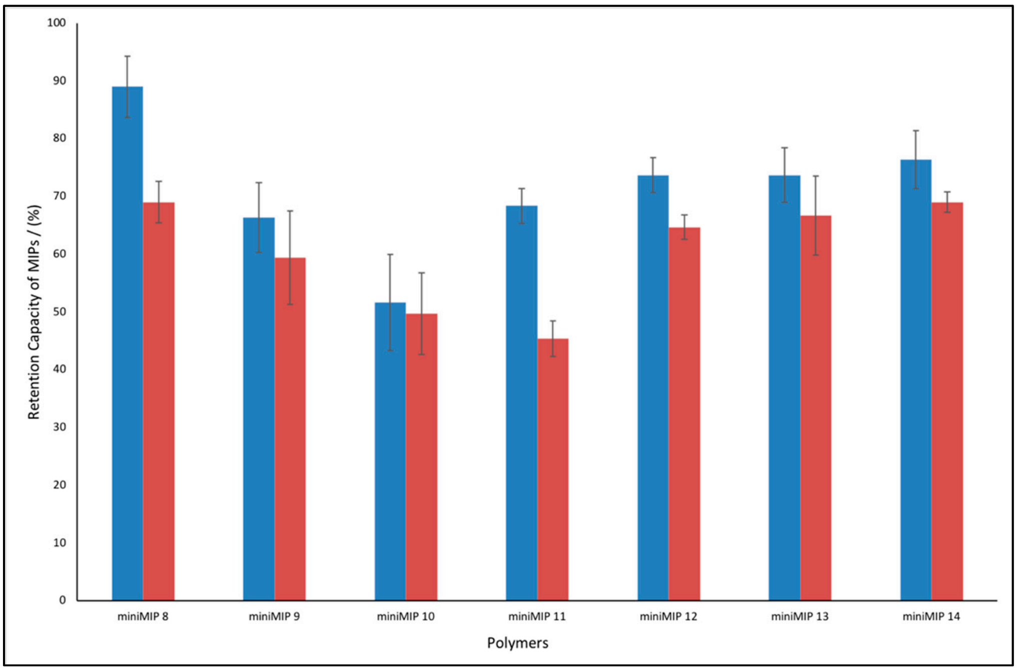

S1. **Figure 9.** PG and BPA retention capacities of miniMIPs made with 4-Vpy like monomer, synthesized according to the experimental design. Error bars represent ±1 standard deviation. Experiments were performed by triplicate.

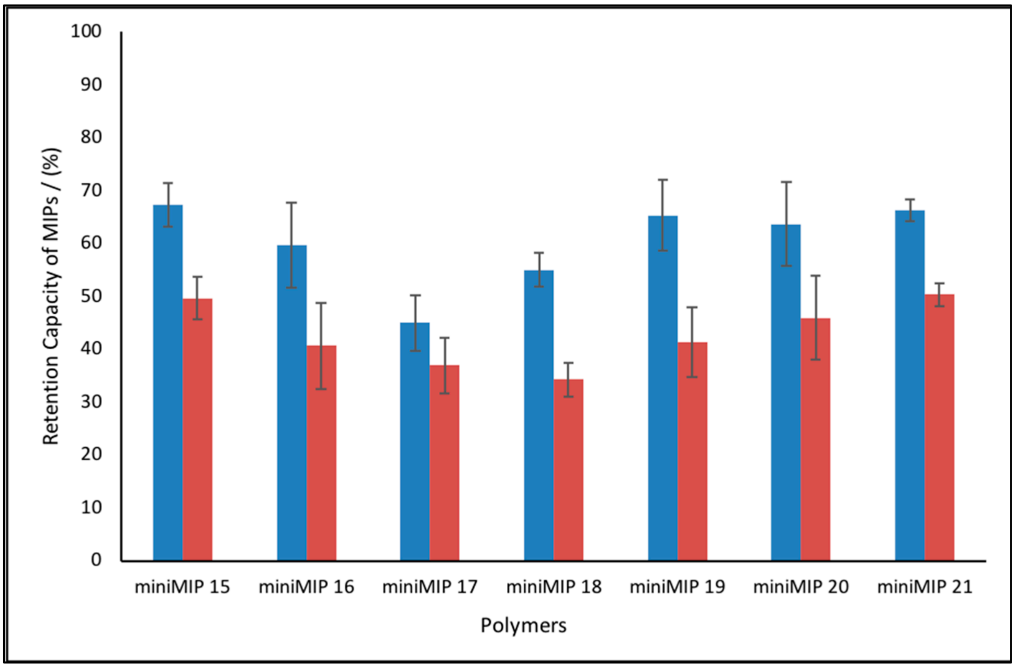

S1. **Figure 10.** PG and BPA retention capacities of miniMIPs made with AAm like monomer, synthesized according to the experimental design. Error bars represent ±1 standard deviation. Experiments were performed by triplicate.

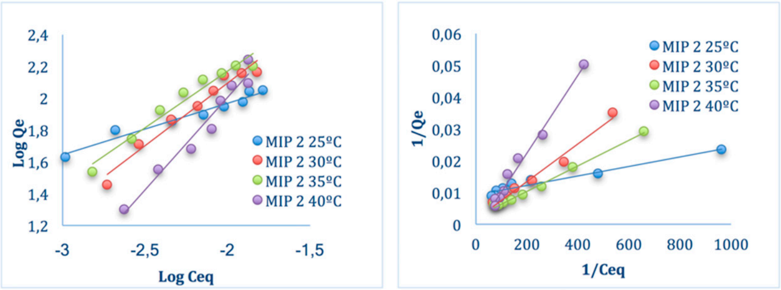

a) b)

S1. **Figure 11** Linearized adsorption isotherms of a) Freundlich and b) Langmuir of MIP 2 for BPA.

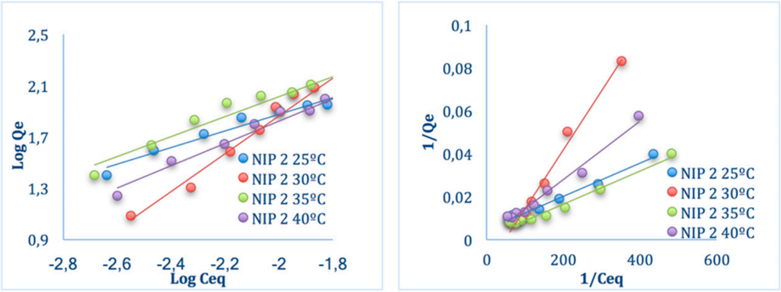

a) b)

S1. **Figure 12.** Linearized adsorption isotherms of a) Freundlich and b) Langmuir of NIP 2 for BPA.

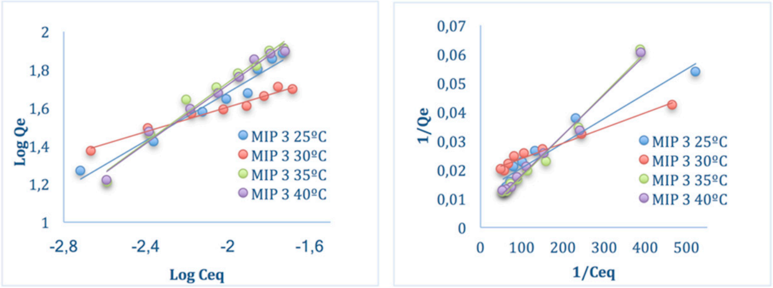

a) b)

S1. **Figure 13.** Linearized adsorption isotherms of a) Freundlich and b) Langmuir of MIP 3 for BPA.

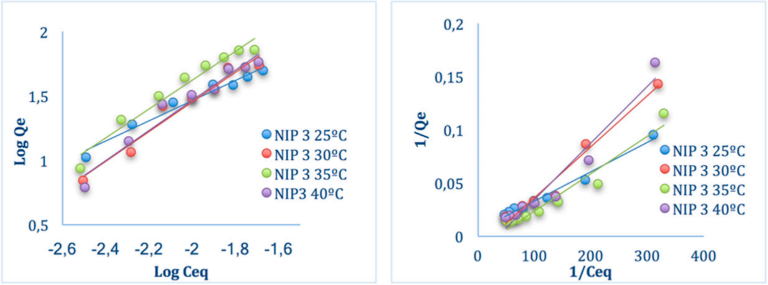

a) b)

S1. **Figure 14**. Linearized adsorption isotherms of a) Freundlich and b) Langmuir of NIP 3 for BPA.

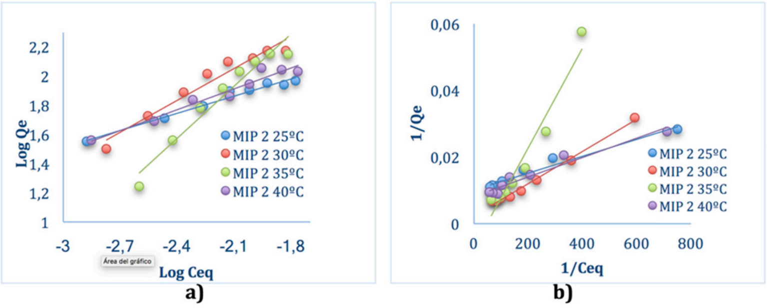

a) b)

S1. **Figure 15.** Linearized adsorption isotherms of a) Freundlich and b) Langmuir of MIP 2 for PG.

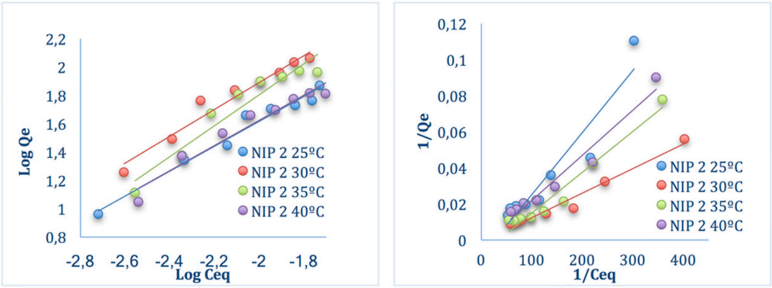

a) b)

S1. **Figure 16.** Linearized adsorption isotherms of a) Freundlich and b) Langmuir of NIP 2 for PG

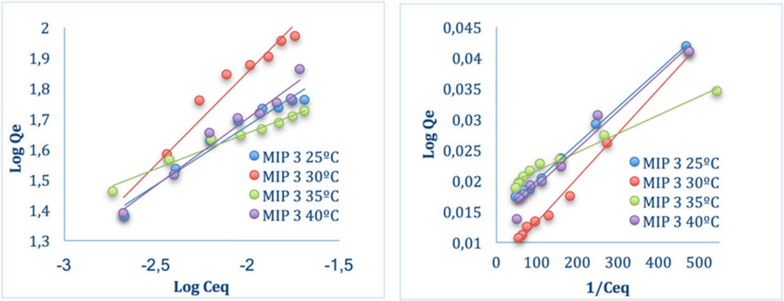

a) b)

S1. **Figure 17.** Linearized adsorption isotherms of a) Freundlich and b) Langmuir of MIP 3 for PG

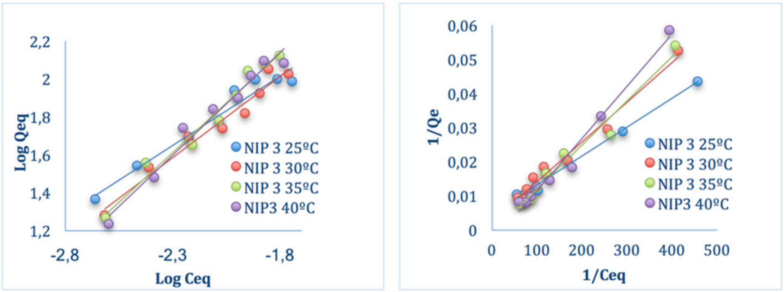

a) b)

S1. **Figure 18.** Linearized adsorption isotherms of a) Freundlich and b) Langmuir of NIP 3 for PG

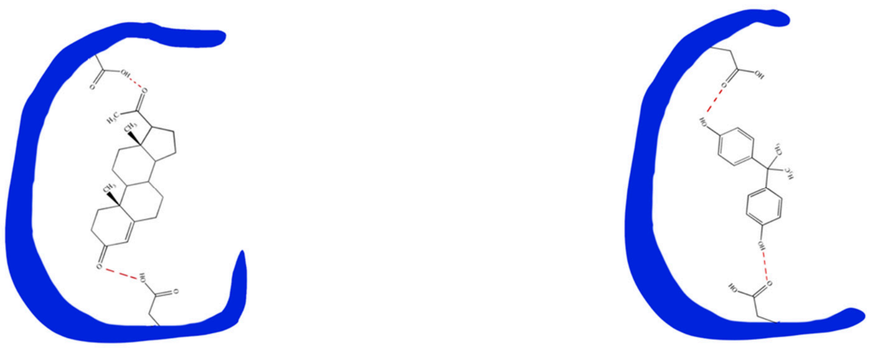

a) b)

S1. **Figure 19.** Schematic representation of the interactions between MIP and a)BPA b)PG.

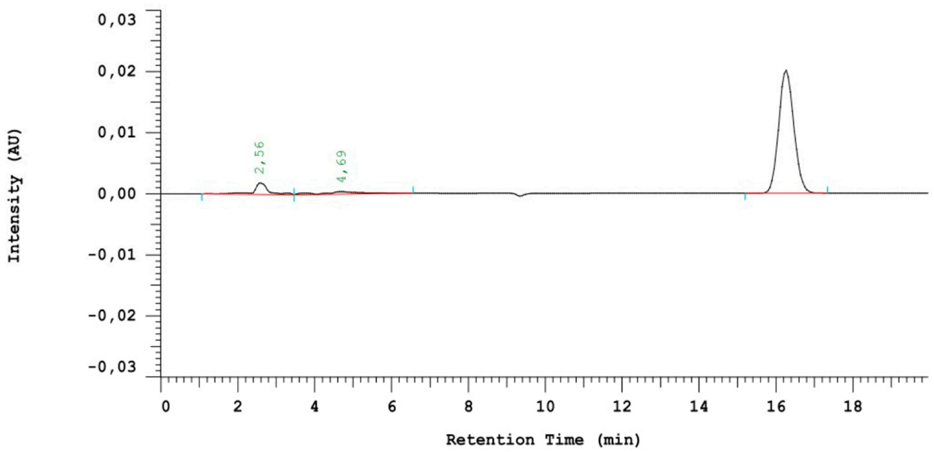

No.	RT	Area	Conc 1	BC
1	2,56	25545	0,000000	BB
2	4,69	15216	0,000000	BB
3	16,27	277392	0,000000	BB
		318153	0,000000	

S1. **Figure 20.** Chromatographic profile of PG before of being contacted with MIP 3, conditions of the experiment are the same than for the retention capacity.

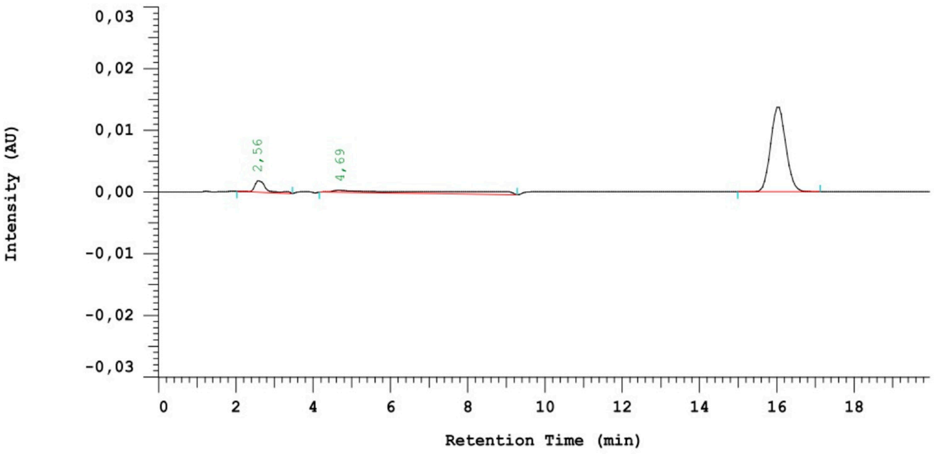

No.	RT	Area	Conc 1	BC
1	2,56	19449	0,000000	BB
2	4,69	40185	0,000000	BB
3	16,05	189286	0,000000	BB
		248920	0,000000	

S1. **Figure 21.** Chromatographic profile of PG after being contacted with NIP 3, conditions of the experiment are the same than for the retention capacity.

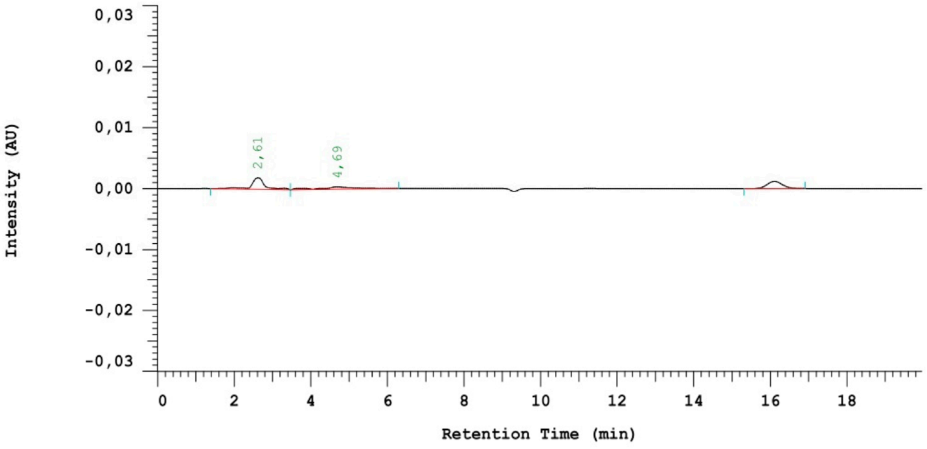

No.	RT	Area	Conc 1	BC
1	2,61	24449	0,000000	BB
2	4,69	13172	0,000000	BB
3	16,11	16822	0,000000	BB
		54443	0,000000	

S1. **Figure 22.** Chromatographic profile of PG after being contacted with MIP 3, conditions of the experiment are the same than for the retention capacity.

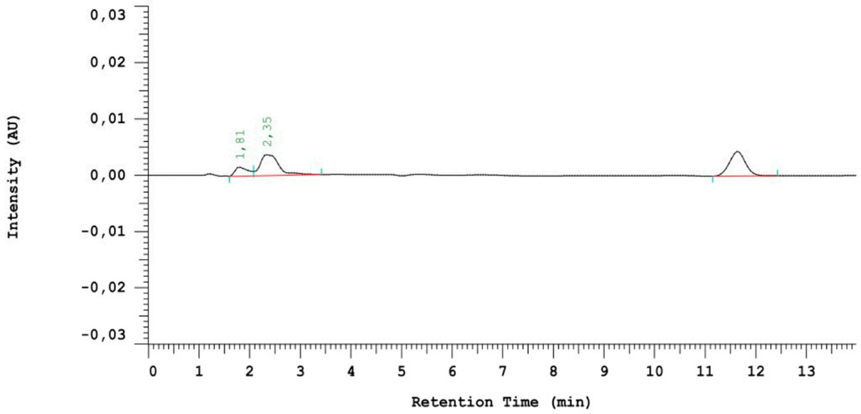

No.	RT	Area	Conc 1	BC
1	1,81	13943	0,000000	BV
2	2,35	48456	0,000000	VB
3	11,63	48358	0,000000	BB
		110757	0,000000	

S1. **Figure 23.** Chromatographic profile of BPA before being contacted with MIP 3, conditions of the experiment are the same than for the retention capacity.

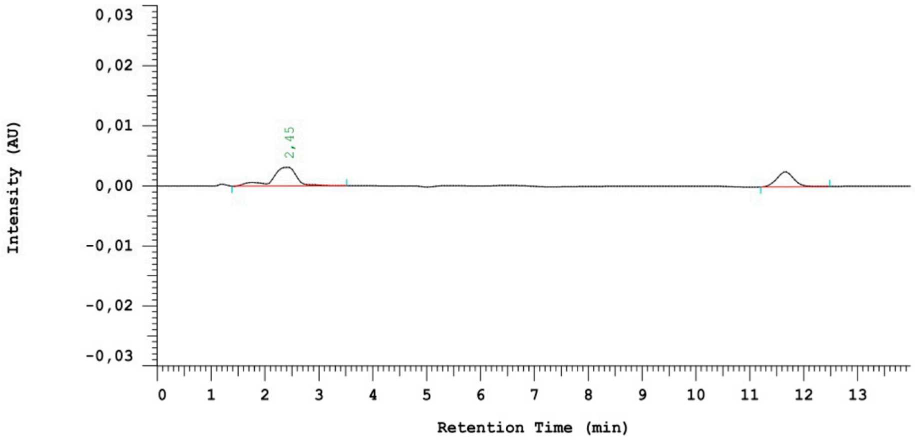

No.	RT	Area	Conc 1	BC
1	2,45	49414	0,000000	BB
2	11,68	27516	0,000000	BB
		76930	0,000000	

S1. **Figure 24.** Chromatographic profile of BPA after being contacted with NIP 3, conditions of the experiment are the same than for the retention capacity.

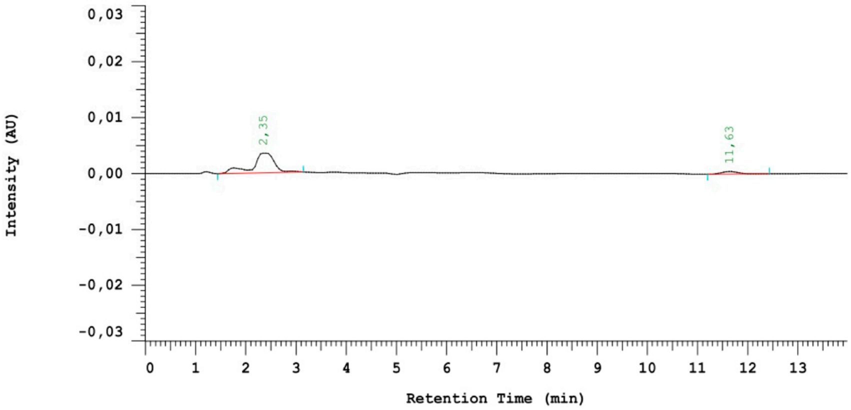

No.	RT	Area	Conc 1	BC
1	2,35	53056	0,000000	BB
2	11,63	5422	0,000000	BB
		58478	0,000000	

S1. **Figure 25.** Chromatographic profile of BPA after being contacted with MIP 3, conditions of the experiment are the same than for the retention capacity.
